# Prediction of Monomer Isomery in Florine: A Workflow Dedicated to Nonribosomal Peptide Discovery

**DOI:** 10.1371/journal.pone.0085667

**Published:** 2014-01-21

**Authors:** Thibault Caradec, Maude Pupin, Aurélien Vanvlassenbroeck, Marie-Dominique Devignes, Malika Smaïl-Tabbone, Philippe Jacques, Valérie Leclère

**Affiliations:** 1 Laboratoire ProBioGEM, Université Lille1- Sciences et Technologies, Villeneuve d’Ascq, France; 2 LIFL, UMR CNRS 8022 Université Lille1 and INRIA Lille Nord Europe, Villeneuve d’Ascq, France; 3 LORIA, CNRS, UMR 7503, Vandoeuvre-les-Nancy, France; 4 LORIA, Université de Lorraine, UMR 7503, Vandoeuvre-les-Nancy, France; 5 INRIA, Villers les Nancy, France; University Paris South, France

## Abstract

Nonribosomal peptides represent a large variety of natural active compounds produced by microorganisms. Due to their specific biosynthesis pathway through large assembly lines called NonRibosomal Peptide Synthetases (NRPSs), they often display complex structures with cycles and branches. Moreover they often contain non proteogenic or modified monomers, such as the D-monomers produced by epimerization. We investigate here some sequence specificities of the condensation (C) and epimerization (E) domains of NRPS that can be used to predict the possible isomeric state (D or L) of each monomer in a putative peptide. We show that C- and E- domains can be divided into 2 sub-regions called Up-Seq and Down-Seq. The Up-Seq region corresponds to an InterPro domain (IPR001242) and is shared by C- and E-domains. The Down-Seq region is specific to the enzymatic activity of the domain. Amino-acid signatures (represented as sequence logos) previously described for complete C-and E-domains have been restricted to the Down-Seq region and amplified thanks to additional sequences. Moreover a new Down-Seq signature has been found for Ct-domains found in fungi and responsible for terminal cyclization of the peptides. The identification of these signatures has been included in a workflow named Florine, aimed to predict nonribosomal peptides from NRPS sequence analyses. In some cases, the prediction of isomery is guided by genus-specific rules. Florine was used on a *Pseudomonas* genome to allow the determination of the type of pyoverdin produced, the update of syringafactin structure and the identification of novel putative products.

## Introduction

Nonribosomal peptides (NRPs) are microbial secondary metabolites which are important pharmaceutical natural products. The unique Norine database contains all available information on the structure and functions of all known NRPs such as the antibiotic penicillin precursor ACV (Norine ID : NOR00006), the immunosuppressive cyclosporin (NOR00033-63) and the biosurfactant surfactin (NOR00211-219. NOR00847-860) [1]. NRPs are built up by huge multimodular enzymatic complexes called NonRibosomal Peptide Synthetases or NRPSs [2]. These megasynthetases can be viewed as assembly lines for peptide synthesis through a step-by-step mechanism. In fact, structure of NRPSs is modular, each module being responsible for the incorporation of one building block or monomer into the growing peptidic chain. The modules are themselves divided into domains catalyzing enzymatic reactions. The main catalytic functions are responsible for the selection and activation of a monomer (Adenylation domain: A), the transfer and tethering of the corresponding adenylate to the NRPS-bound 4′-phosphopantetheinyl cofactor (Thiolation domain: T), peptide bond formation (Condensation domain: C), and the release of the peptide, sometimes accompanied by its cyclization (Thioesterase domain: Te, terminating the NRPS). Because the C-domain is generally absent from initiation module, the general architecture for NRPS modules is schematized as {(C)-A-T} (the “C” in brackets means present or absent depending on the type of module).

The basic function of a condensation domain is to catalyse the peptide bond formation between two amino-acids linked to their thiolation domains. In the past, various types of C-domains have been distinguished, mostly by multiple sequence alignments combined with phylogenetic studies [3]–[5]. The ^L^C_L_ domains catalyse the condensation between two L-monomers, the dual C/E-domains are capable of both epimerization and condensation leading to a bond between a D- and an L-monomer. The ^D^C_L_ domains also catalyse the condensation between a D- and an L-monomer but it is not responsible for epimerization. When present at the beginning of a NRPS, the C-starter domains catalyse the condensation of a lipid moiety or salicylic acid derivative onto the first monomer of the peptide chain. Finally, at the last position in fungal NRPSs, Ct-domains are responsible for both the release and cyclization of the peptide [6].

A key structural feature of nonribosomal compounds is the modification of some of their building blocks during biosynthesis. Additional domains, modifying the monomers during their incorporation, are sometimes present. Among them, the most frequent is the Epimerization domain (E), which modifies an L-monomer into its D-isomer on the α-carbon.

Analysis of numerous NRPSs in relation with their products has shown that different microorganisms have developed different strategies to incorporate D-monomers within their active peptides. In a wide range of NRPS an E-domain is located directly after the T-domain, leading to a particular module architecture {(C)-A-T-E} instead of the usual {(C)-A-T} described above. Cyclic lipopeptides (CLPs) produced by species belonging to *Bacillus* and *Pseudomonas* genera include a large proportion of D-monomers [7]. All *Bacillus* CLP synthetases harbour E-domains in the modules corresponding to each D-monomer but, surprisingly, no E-domain has been detected in *Pseudomonas* CLP synthetases. In these assembly lines, epimerization occurs through the dual C/E-domain mentioned above. Such C/E-domains epimerize the activated monomer linked to its cofactor on the T-domain immediately preceding them. Therefore, they are found in the elongation modules corresponding to the following monomer, which can thus be displayed as {C/E-A-T}. Since their initial finding in the arthrofactin (NOR00343) synthetase of *Pseudomonas* sp.MIS38, the presence of dual C/E-domains has been generalized to all CLP synthetases studied so far in Pseudomonads [3], [8], [9]. Interestingly, this particular strategy for monomer isomerisation co-exists in *Pseudomonas* genomes with the more frequent strategy involving E-domains as observed for pyoverdin synthesis.

A third strategy for D-monomer integration into NRPs relies on the recent observation that several adenylation domains are able to directly activate D-monomers both in fungi and bacteria [10], [11]. This implies that natural L-monomers have been isomerized by racemases acting in *trans*. Until now, only examples of D-alanine loaded by A-domains have been described. The D-Ala is provided by Alanine racemase or Alr (EC 5.1.1.1) that is encoded by a separate gene [11].

An urgent challenge today is to discover new natural drugs to tackle emerging pathogens and to obtain efficient anti-tumoral compounds. Bioinformatics approaches are largely considered for this purpose as a way to save time during the screening of such molecules. Regarding nonribosomal peptide synthesis, specific tools have been developed for predicting the organization of NRPS modules in terms of domains and the nature of the monomers incorporated by A-domains [12]–[16]. In parallel, analysis of the Norine database content has established some relationships between the monomeric composition of nonribosomal peptides and their probable biological activity [17], [18]. However, until now, less attention has been paid to the prediction of epimerization although D-isomery can provide resistance to proteolysis, and stereo-chemical constraints are sometimes mandatory for cyclization of the peptides as for tyrocidin antibiotic (NOR00298-301) [19], or which are necessary for biological activities as for surfactin (NOR00211-219, NOR00847-860) [20].

The work presented herein describes a new strategy for identifying C- and E-domain sub-types leading to the prediction of monomer isomery. As epimerization is essential for architectural diversity of the NRPs, its prediction was considered as a key step of the Florine workflow that was developed to improve structural prediction of peptides from NRPS sequence analysis.

## Materials and Methods

### NRP and NRPS Data

Sequences were extracted from universal databases for DNA or protein sequences [21], [22] and from the Norine database for nonribosomal peptides [08]. The Norine identifiers of NRPs (NOR00XXX) are specified each time the peptides appear in the text. Some NRPS sequences were obtained through links to UniProt from the Norine database.

### Annotation of NRPSs

The catalytic domains occurring in NRPS proteins were identified with widely used tools such as InterProScan at the EBI [23] and Conserved Domain Search Service (CDSS) at the NCBI [24], and with tools specifically dedicated to PKSs (polyketide synthases) and NRPSs (Table 1). We mainly combined the results from five bioinformatics tools dedicated to NRPSs and described in Table 1 (for reviews see [13], [25], [26]). We did not use ClustScan [27] for this study because it is mainly dedicated to PKSs and does not predict the monomers selected by NRPS A-domains. We also did not use NP.searcher [28] because it only gives a list of monomers as output. Some details about the quality of the prediction and the start positions of the A-domains are given in the result log file, but these data are difficult to find, especially for biologist who might lack strong computer science skills. All of the tools that we used are freely available on-line (see URLs in the reference list of Table 1).

**Table 1:** Main features of the tools used in this study to analyse NRPS.

	Input	Enzymes	Domains*	Product format	Ref
**NRPS-PKS**	Protein	NRPS, PKS	All types	Monomers	[12]
**NRPSPredictor2**	Protein	NRPS	A domains	Monomers	[14], [15]
**PKS/NRPS analysis**	Protein	NRPS, PKS	All types	Monomers	[13]
**antiSMASH**	ADN/protein	NRPS, PKS, other	All types	Monomer list, SMILES**	[16]
**NaPDoS** ***	ADN/protein	NRPS	KS/C families	none	[5]

* All types of domains means that the tool outputs all the known domains for the enzymes they cover. “A domains” is for adenylation domains; “KS” for ketosynthase and “C” for condensation domains.

** SMILES (*simplified molecular-input line-entry system*) format is a string representation of chemical structures.

*** NaPDoS works better with one domain at a time.

In summary, the global NRPS architecture was predicted using NRPS-PKS (12), PKS/NRPS analysis [13], and antiSMASH [16], [29] programs. In addition, NaPDos was used to determine C-domain types (5). Monomer prediction based on A-domain specificity was conducted with the NRPS-PKS, PKS/NRPS analysis and antiSMASH programs mentioned above, together with the NRPSpredictor2 program [14], [15]. Finally, Norine [1] was used to search for known peptides having similar structure to the predicted peptides.

### Study of Domain Sub-types and Creation of Weblogos

To study the specificity of the domains described in this article, we performed multiple alignments using the MUSCLE program [30]. Sequence logos were designed using Weblogo [31]. The set of sequences used for this study is partially derived from the panel used by Rausch *et al.* [4] who performed sequence logos extraction on 442 sequences of full-length C-domains corresponding to C-starter, ^L^C_L_, ^D^C_L_, and dual C/E domains. Because Rausch *et al*. [4] did not consider sequences when the complete genome was not available, additional sequences were imported from our studies of NRPSs in *Pseudomonas* [9], *Burkholderia* (personnal data), *Bacillus* [32] and fungi [6] in order to enrich the dataset. Thus, 153 sequences were added (9 Cstarter, 60 ^L^C_L_, 19 ^D^C_L_, 31 E and 34 dual C/E)(Table S1). Except for *Burkholderia* synthetases, the products(s) of added NRPSs are known and specified in the table S1.

NRPS domains were identified based on their InterPro ID (IPR000873, IPR009081, IPR001242, and IPR001031 for A, T, C and Te domains, respectively). Then, the different sub-types of C-domains were classified according to their neighbourhood. Indeed, if the sequence upstream of a C-domain was less than 50 aa in length, it was considered as a C-starter. If a tandem of C-domains was observed, the first one was considered as an epimerization domain and the second one as a ^D^C_L_. Remaining domains were classified into ^L^C_L_ group. Finally, a manual cleaning was performed and the classification of domains was checked mainly based on our knowledge about the products. Dual C/E and Ct were classified according to their known activity.

The Down-Seq regions are always delimited between the end of an IPR001242 C-domain and the start of the following domain (A or C). For each group, alignments were performed independently on the Down-Seq regions, and weblogos were designed.

## Results and Discussion

### Comparison of D-monomer Occurrence in Ribosomal- and Nonribosomal- Peptides

**Figure 1:**
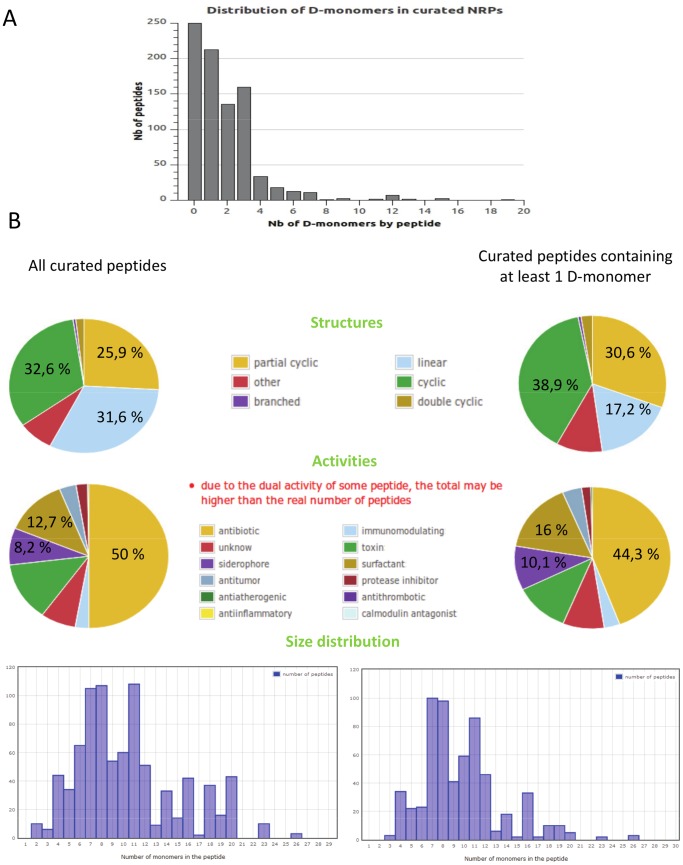
Comparison of D-monomer occurrence within ribosomal and nonribosomal peptides. The data are extracted from Norine database. **A**: Distribution of D-monomers in curated NRPs (Nb : number), **B**: Comparison of structures, activities and size distribution between all peptides and those containing at least 1 D-monomer. For the structures, only the 3 major percentages are indicated (cyclic, partial cyclic and linear). Only percentages related to the main activities studied in the paper are indicated (antibiotic, surfactant and siderophore).

The distribution of D-monomers in NRPs has been studied in the Norine database. We have found 1920 D-isomers among the 11,206 monomers composing the 1164 nonribosomal peptides of Norine, distributed across 213 families. This frequency of 1,7.10^−1^ is very high compared to the 5.10^−6^ frequency found in proteins and peptides of the SwissProt database [33]. Moreover, near 80% of the Norine peptides harbour at least one monomer in D-configuration and among them 77% harbour more than one (Fig. 1A). Thus, the occurrence of D-monomers within a peptide remains a good clue for predicting a nonribosomal biosynthesis, even though some examples of epimerization through post-translational modification have been described recently in ribosomal peptides [34]. According to this criterion, some of the peptides hosted in the Norine database are referred to as “putative”, due to the presence of D-isomers, which means hypothetical nonribosomal origin because no synthetase is known. An example is the gratisin (NOR00657), a cyclic antibiotic produced by *Brevibacillus brevis* displaying the following circular sequence [Val,Orn,Leu,D-Phe,Pro,D-Tyr,Val,Orn,Leu,D-Phe,Pro,D-Tyr] and for which no NRPS gene has been identified yet. In this particular case the presumption for NRPS synthesis is also supported by the presence of the non-proteogenic monomer ornithin.

Next, we compared the structures and activities displayed by the Norine peptides containing D-monomers with those displayed by the complete set of Norine peptides (Fig. 1B). All possible structures, activities and sizes are encountered in both groups. However, the occurrence of D-monomers is correlated to a lower ratio of linear peptides vs cyclic and partially cyclic structures. Independently of the presence of D-monomer(s), the antibiotic activity, which is mainly researched during new drug prospection, is overrepresented. Therefore the prediction of epimerized monomers within a peptide appears an important step of a workflow dedicated to the discovery of new active peptides.

### Condensation and Epimerization Domains are Members of the Same Super-family

**Figure 2:**
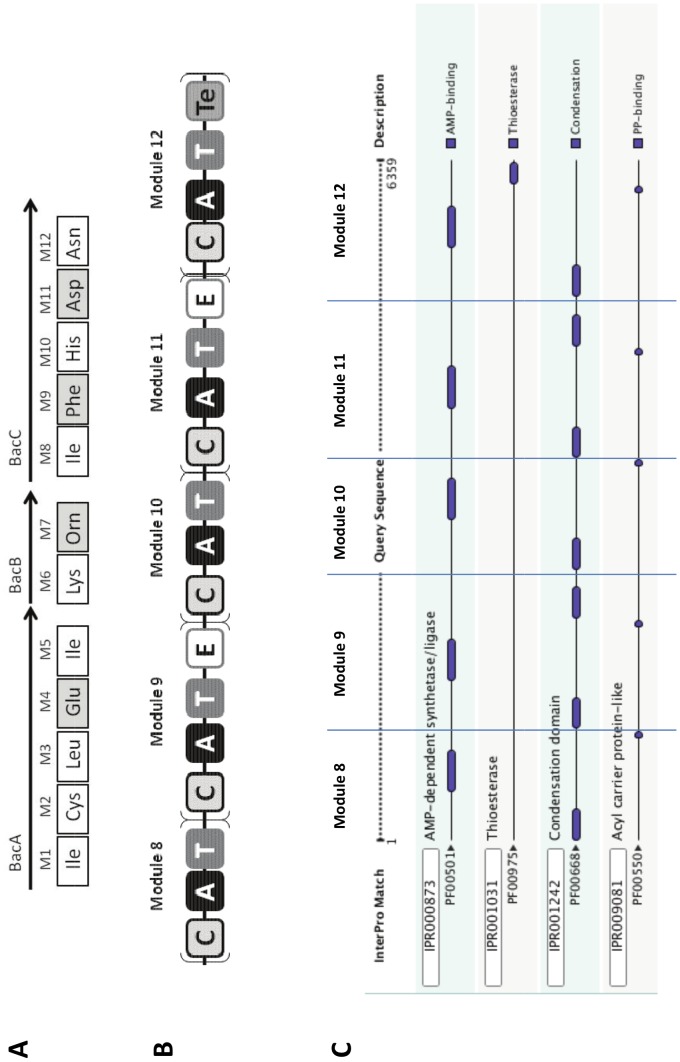
Architecture of the bacitracin synthetase. **A**: Modular organization of the proteins constituting the complete bacitracin synthetase. The names of the proteins are mentioned above the arrows. The monomer activated by each module (M1 to M12) is indicated in the square under the corresponding module, the squares are white for L-monomers and grey for D-monomers. **B**: Domain architecture of BacC protein : schematic representation inspired from various NRPS analysis tools, A: adenylation domain, C: condensation domain, T: thiolation domain, E: epimerization domain, Te: thioesterase domain. **C**: Results from InterProScan analysis of BacC protein, the separation between modules has been added and is represented by blue lines.

**Figure 3:**
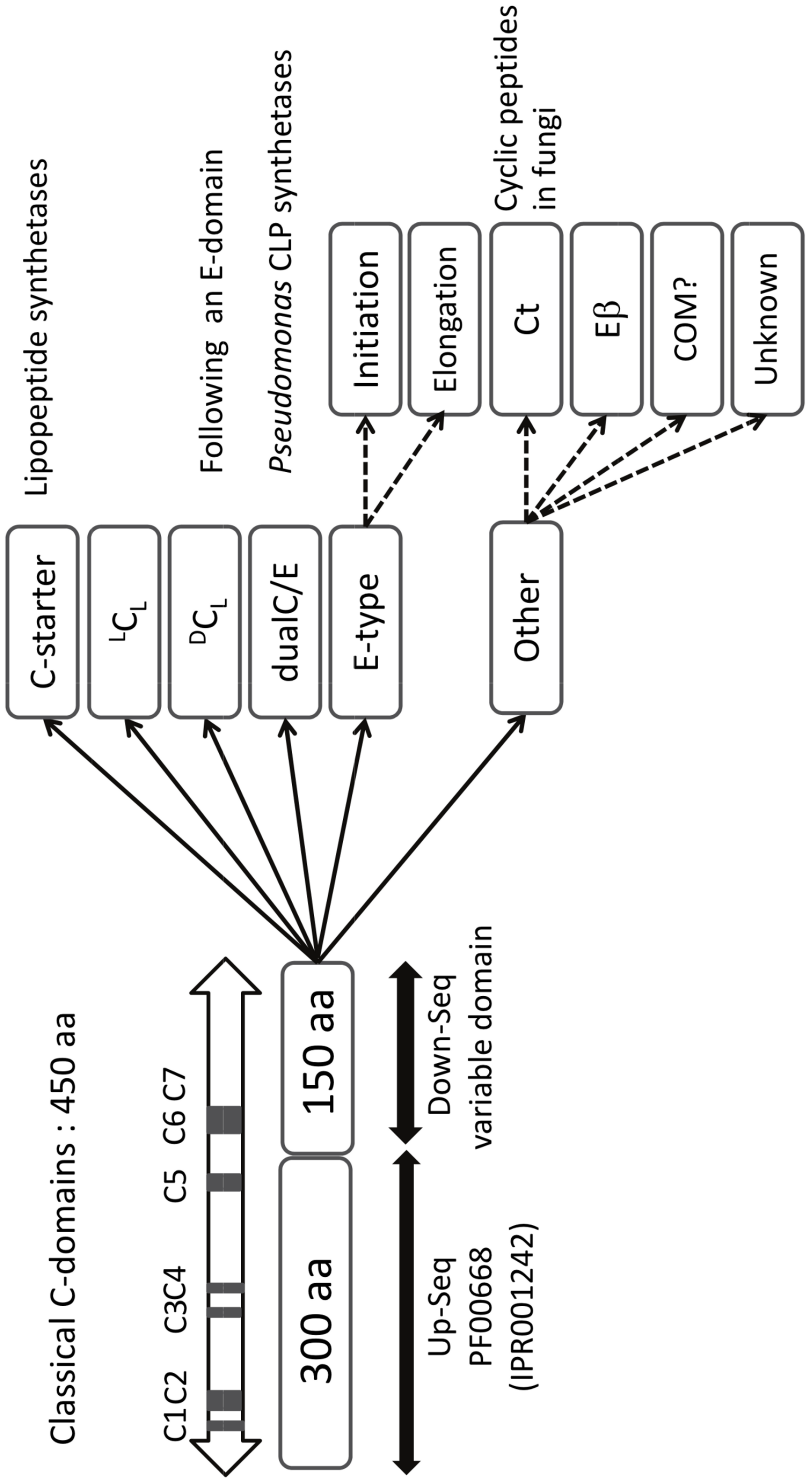
Up-Seq and Down-Seq regions in condensation and epimerization domains. aa : aminoacids.

In NRPSs, D-isomer incorporation most frequently relies on the presence of an additional domain, the E-domain, that catalyzes the epimerization reaction. This E-domain is generally located downstream of the A and T domains involved in the monomer activation. We have compared the detection of such E-domains, using dedicated or common tools, in the protein sequence of the three NRPSs (BacA, BacB and BacC) responsible for the synthesis of the well-known antibiotic bacitracin (NOR00018-22, NOR00913-923) [35] produced by *Bacillus licheniformis.* This active compound contains 4 D-isomers out of its 12 monomers (Fig. 2A, grey boxes). For each D-monomer an epimerization domain (E) is present in the corresponding module of the synthetase: modules 4 in BacA protein, 7 in BacB protein, 9 and 11 in BacC protein (Fig. 2B). All four E-domains were well detected by dedicated tools such as the NRPS-PKS [12], PKS/NRPS analysis [13] and antiSMASH 2.0 [29] programs, but not by the web tool NaPDos when the complete sequence of each NRPS was considered [5]. Surprisingly, the tools based on domain databases such as InterPro and CDD reveal the same Pfam domain PF00668 (or IPR 001242) in both E- and C-domains (Fig. 2C). In fact, the HHxxxDG signature contained in this PF00668 domain has been shown to be essential for both condensation and epimerization activities [36]. In epimerization domains, the second Histidine residue (H) is involved in proton abstraction and re-addition on the Cα concerned by epimerization. This residue is also involved in the nucleophilic attack of the acyl N-terminus in the condensation mechanism. The lengths of the C- and E-domains extracted from specific tools are quite similar (450 amino acids in average) preventing discrimination by this single criterion. A systematic InterProScan analysis carried out on 137 NRPS modules revealed that a sequence of 165±10 amino-acids always separates the Pfam domain PF00668 (IPR001242) from the following one, being an A- or a C-domain depending on studying C- or E-domains, respectively. Multiple sequence alignments of C- and E-domains clearly showed that the E- and C-domains can be divided in two regions. The first one covering the 300 first amino acids is conserved between E- and C-domains and is called here Up-Seq region. The second one spanning the remaining 150 amino acids appears to be specific for the catalytic activity of the domain and is called here Down-Seq region (Fig. 3). The Up-Seq region corresponds to the Pfam motif PF00668 (IPR001242) and contains the HHxxxDG active site motif [37]. It encompasses the highly conserved core motifs C1 to C5 for condensation and E1 to E5 for epimerization as defined by Marahiel *et al.* in 1997 [36], while C6, C7 and E6, E7 conserved motifs also defined by Marahiel *et al.* are located within the Down-Seq region. Interestingly, an InterPro domain (IPR009081, TIGR01720) is detected in the Down-Seq region of E-domains when the InterProScan search is performed using the TIGR domain database. This short 171-aa domain is described as a non-ribosomal peptide synthase domain, located downstream a condensation domain. However to our knowledge it has never been associated with epimerization domains. Moreover, no counterpart of this domain exists in the TIGR domain database for the Down-Seq region of C-domains.

We have compared this organization in 2 domains with the 3-D structure of a condensation domain [38]. This unique structure (*Vibrio cholera* VibH condensation enzyme, PDB1l5a) can be considered as a pseudodimer with N- and C-terminal structural domains facing each other. However the separation between these two structural domains occurs between two α-helices (α5 and α6) located inside the PF00668 domain after the conserved HHxxDG motif. Thus more than one third of the PF00668 domain is actually part of the C-terminal structural domain [39]. Interestingly the N-terminal structural domain borrows a strand from the C-terminal domain (β12) to complete a β-sheet. This strand is located downstream of the PF00668 domain, in the Down-Seq region of this C-domain. Thus both Up-Seq and Down-seq regions are involved in both N- and C- terminal structural domains.

From a functional point of view, the PF00668 (Up-Seq region) contains the solvent channel that drives the extension of the pantetheinyl arm to present the substrates for catalysis and the Down-Seq region forms a part of the structure that interacts with the nascent peptide. We can hypothesize that this region may play a differentiating role between various C- and E- domains.

### A Variety of Condensation and Epimerization Domains Related to Down-Seq Region Diversity

**Figure 4:**
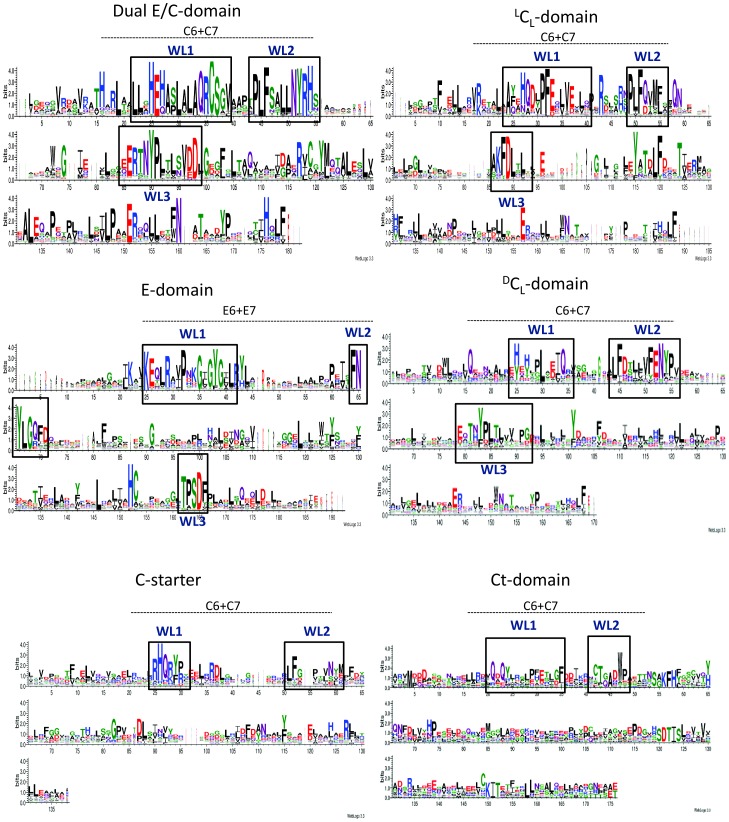
Figure 4. WebLogo signatures for E- and C-domains. C6,C7 and E6, E7 (signatures 6 and 7 for condensation and epimerisation domains, respectively) are highlighted by the dotted lines. The Weblogos (WL) numbered WL1, WL2 and WL3 are mentioned in blue and the corresponding new signatures are surrounded by black squares.

In the super-family of condensation and epimerization domains, several sub-groups have been defined which include C-starter,^ L^C_L_, ^D^C_L_, E, dual C/E, and Ct (in fungi). Without exception, we have found that all of them have the same architecture with a well-conserved common Up-Seq region, tagged by the presence of a PF00668 domain, and a differentiating Down-Seq region (Fig. 3). We therefore focused our attention on the Down-Seq region in order to detect signatures corresponding to each type of function. Multiple alignments were thus performed on the Down-Seq regions taken from each sub-group of C- and E-sequences. Because sequences spanning about 150 amino acids are too short to design relevant phylogenetic trees, we decided to transform each multiple alignment into a sequence logo using the WebLogo (WL) application [31]. We were able to highlight 3 signatures for each of ^L^C_L_, ^D^C_L_, dual C/E and E sub-group, referred to as WL1, WL2 and WL3 (Fig. 4). These WLs are specific of each ^L^C_L_, ^D^C_L_ and dual C/E sub-group. While the WL1 and WL2 signatures nicely match with the signature logos published by Rausch *et al.* [4] for motifs C6+C7,the WL3 signature is new, located further downstream in the sequences. To exemplify the use of the three WL signatures, the identification of each type of C- and E-domains in bacitracin synthetase BacA, BacB and BacC proteins, in syringafactin (NOR01075-80) synthetase SyfA and SyfB proteins, and in kurstakin synthetase KrsC protein, is presented in Supplementary material (Figure S1). As in all other NRPSs tested, the various types of C- and E-domains can be correctly predicted by the presence in their Down-Seq region of the type-specific WL1, WL2 and WL3 signatures and by the lack of any other signatures.

For the C-starter domains we have aligned 20 Down-Seq regions of NRPS that mainly direct the synthesis of cyclic lipopeptides in *Bacillus* and *Pseudomonas* species. We have identified two signatures located at similar positions as the WL1 and WL2 signatures described above and which overlap nicely with the signatures published by Rausch *et al*. [4] (Fig. 4).

Finally we have also searched for signatures to identify the more recently described Ct-domains that can be found at the C-terminus of fungal NRPS and lead to peptide cyclization [6]. Two WL signatures are found (Fig. 4) but these are not very strong because the number of available sequences related to cyclic peptides is still weak. Nevertheless, no WL signature specific for the other types of C- and E-domains was detected within the Down-Seq region of fungal Ct-domains. This demonstrates that the Ct domains display their own signatures which will be defined more accurately when more sequences become available.

Recently, a novel type of epimerization domain was identified in the lysobactin synthetase from *Lysobacter* sp.ATCC 53042 [40]. This unusual epimerization domain (called Eβ) is located directly after the condensation domain in module 8 that displays the {C-Eβ-A-T} structure. Indeed, this domain acts as a side-chain epimerization domain. As for classical E-domains, the PF00668 (IPR001242) condensation domain is recognized by InterProScan in the Up-Seq region of this Eβ−domain. This Pfam domain is followed by a Down-Seq region specific of the Eβ-domain (Fig. 3). Because only one example of such Eβ-domain is known, it is not possible to extract any signature within the Down-Seq region. However, it is interesting to note that none of the signatures established for either C-starter,^ L^C_L_, ^D^C_L_, dual C/E, or E-domains was encountered in this sequence.

In fact, we believe that focusing on the Down-Seq signatures identified here, and thus reducing the length of analyzed sequences, may be sufficient to characterize NRPS domains in incomplete sequences for example from partial contigs or unfinished draft genomes.

### Florine : a Workflow for Structure Prediction of NRPs

**Figure 5:**
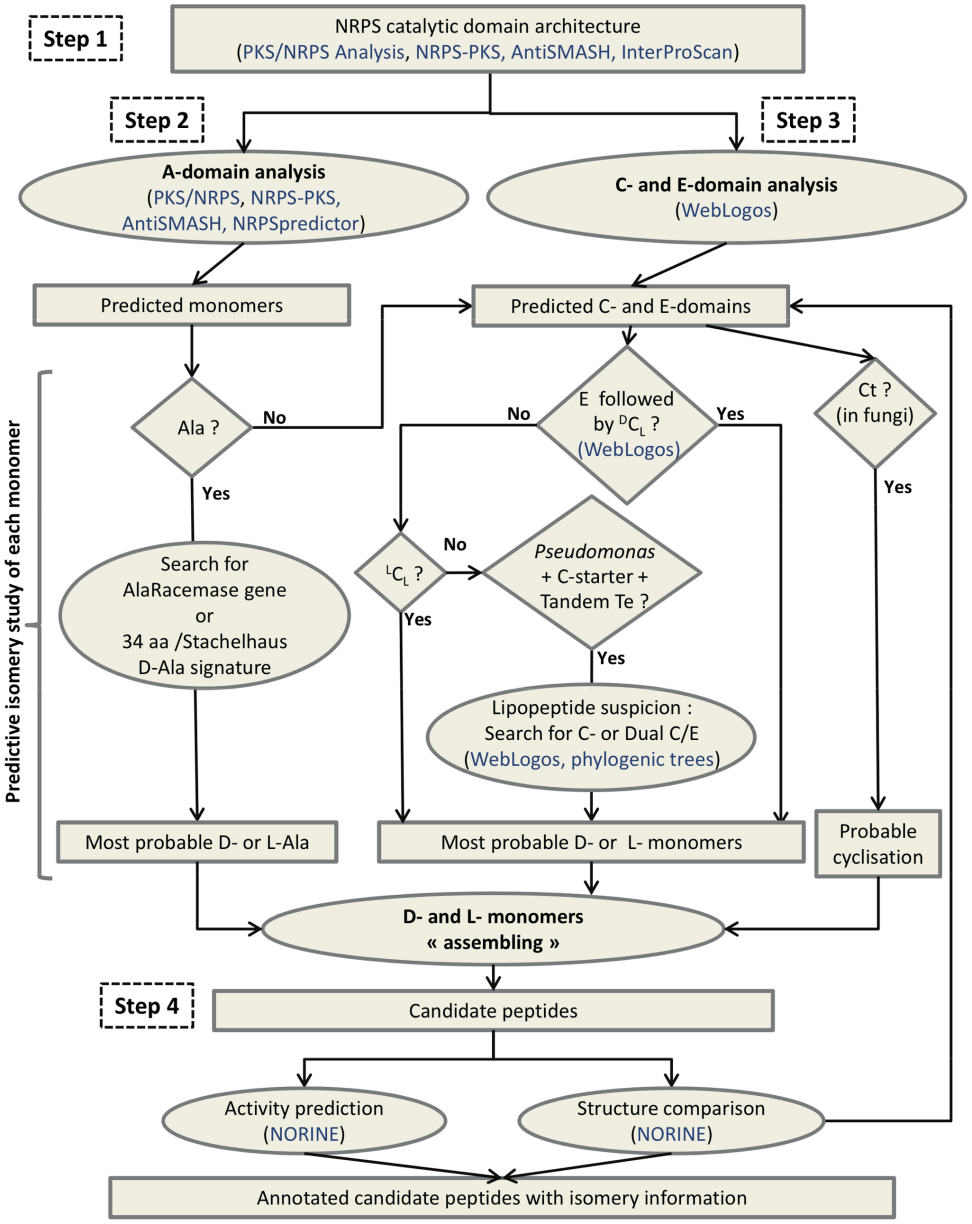
Florine : a workflow dedicated to structure prediction of nonribosomal peptides. Squared boxes are for data (results of bioinformatic processes) and ovals for data processing. Diamond-shaped boxes indicate questions with yes or no answer, bioinformatic tools and databases are mentioned in blue.

The workflow called Florine, including isomery determination, was developed for structure prediction of NRPs (Fig. 5). The main steps are 1) the identification of putative NRPSs from genomic data by the determination of their typical modular organization involving C, A, T and Te domains, 2) the determination of adenylation domain specificity for the prediction of incorporated monomers, 3) the analysis of C- and E-domains to get the best prediction of the isomeric status of each monomer, 4) the design and characterization of the peptide and its comparison with existing peptides. In step 1, the UniProt-KB database and the universal tool InterProScan are used in conjunction with the three specific tools already mentioned (NRPS-PKS, PKS/NRPS Analysis and AntiSMASH) for defining the modular organization of a putative NRPS. It seems most efficient to combine the results obtained from several programs as their efficiency may vary from one synthetase to the other, because some domains are difficult to predict.

In step 2, the most probable monomers can be predicted from each A-domain sequence by using the Stachelhaus code and Transductive Support Vector Machines (TSVM) technology as proposed by NRPSpredictor [15] in addition to the strategy of Minowa *et al.* [41] as implemented by antiSMASH [16]. In this step however, one should keep in mind that sequence specificities may be different between bacteria and fungi. Moreover, for some monomers, A-domain sequences are not yet sufficiently numerous for defining specific signatures. Concerning Ala monomer prediction, it can be checked further whether the A-domain signature predicts an L- or a D-isomer. The D-isomer prediction can be supported by the presence of an Alr gene in the genome.

In step 3 all domains mentioned as C or E have to be analysed to determine the putative isomery of monomers other than Ala. The different types of C-domains (dual C/E, ^L^C_L_, ^D^C_L_, C-starter, Ct) have to be identified by the signatures represented by the WebLogos at WL1, WL2 and WL3 positions within the Down-Seq region (Table S2). Normally the E-domains are followed by a ^D^C_L_ domain present at the beginning of next module, which is responsible for the junction between the D-monomer isomerized by the E-domain, and the following L-monomer. In some cases, it can be observed that the latter monomer also turns into a D-monomer but this occurs at the next elongation step if an E-domain is present in its module.

It should be noted that step 3 largely depends on the strain (fungi vs bacteria, *Pseudomonas* vs others), and that this information can direct the type of analysis to perform. For *Pseudomonas* if a cyclic lipopeptide synthesis is suspected (presence of a C-starter in the first module, presence of a tandem of Te-domains ending the NRPS, and lack of E-domains), the occurrence of dual C/E-domains has to be searched among all C-domains. For fungal NRPSs, the presence of a Ct as the last domain favours the prediction for a cyclic peptide.

Finally, in step 4, the comparison of the predicted NRP structure with all known NRPs is performed to complete the prediction. This step is easy to carry out using the Norine database tools which offer structure search functionalities. For example an editor allows the design of peptides with the possibility of mentioning, at each position several monomers (including D- or L- configurations) [1], [17], [42]. The system then returns all the similar peptides present in the database. This can provide for a given peptide a putative family in which peptides generally differ on the nature of the monomers but not on their isomery.

Step 3 is the central feature of the Florine workflow. For the first time, discrimination between ^L^C_L_ and ^D^C_L_ is possible to support the D-prediction originally based on the presence or absence of E-domains., Even if the the NRPS/PKS and PKS/NRPS Analysis tools sometimes mention the stereoisomery of the predicted monomer, it should not be considered because it cannot been deduced from Stachelhaus code. For example, a D-Tyr is predicted by the NRPS/PKS tool as the monomer incorporated by the second module of the *Bacillus thuringiensis* kurstakin synthetase C (KrsC), whereas the PKS/NRPS Analysis tool does not return any prediction for that module. In fact, one D-monomer is present in Kurstakin but the Florine workflow identifies it as a D-Gln associated with the third module of the NRPS [43], thanks to the presence of an E-domain in this module, followed by a ^D^C_L_-domain in the next module, as identified using their respective WL signatures (Table S2).

In step 3, several possibilities may be proposed, depending on the context or on the producing strain. It is also important to note that two epimerization strategies may co-exist in a single microorganism as in *Pseudomonas* where dual C/E-domains are found in CLP synthetases together with pairs of E- and ^D^C_L_-domains in other NRPS. Another example is fusaricidin synthetase in which pairs of E- and ^D^C_L_-domains co-exist with an A-domain directly loading a D-Ala previously epimerized by a racemase [44].

### Identification of New Peptides from Genomic Data of *Pseudomonas*

The Florine workflow which takes advantage of all WL signatures presented above was applied to all available *Pseudomonas* genomes. These genomes were searched to identify NRPS and a special attention was paid to the prediction of the isomeric status of each monomer, to complete the potential structure of the identified peptides. We describe here the results obtained with the genome of the phytopathogenic *Ps. syringae* pathovar *tomato* DC3000 (taxid 223283), which was found to contain several NRPS genes involved in the synthesis of both known and unknown peptides. The complete genome of 6.5 Mb consists of one chromosome and two plasmids which together encode 5763 ORFs [45]. This strain is known to produce 3 distinct siderophores (salicylic acid, yersiniabactin and one pyoverdin)46] and cyclic lipopeptides belonging to the syringafactin family [47]. Using a keyword search strategy among automatic gene annotations combined with BLAST analyses performed with the *Bacillus* MycB protein as a query (taxid 223283) we have identified 12 putative NRPS genes distributed over five clusters in the chromosome (NC_004578) (Table 2). Similar results were obtained with the antiSMASH 2.0 [29], except that the tool returns 7 clusters among which 2 are erroneous, i.e; they do not have a NRPS domain.

**Table 2:** Clusters of NRPS genes identified in the genome of *Ps. Syringae* pv. *tomato* DC3000.

protein id	domains	peptide	ref
AA056104	T-Cy-A-T	Yersiniabactin	46
AA056106	PKS-Cy-T-Te		
NP_791957	T-^L^C_L_-A-T-^L^C_L_-A-T-E-^D^C_L_-A-T	pyoverdin 19310	46
NP_791969	^L^C_L_-A-T	NOR00199	this study
NP-791970	^L^C_L_-A-T-E-^D^C_L_-A-T		
NP_791971	^L^C_L_-A-T-^L^C_L_-A-T		
NP_791972	^L^C_L_-A-T-E-^D^C_L_-A-T-Te		
NP_792633	C_starter_-A-T-C/E-A-T-C/E-A-T	syringafactin	47
NP_792634	C/E-A-T-^L^C_L_-A-T-C/E-A-T-^L^C_L_-A-T-C/E-A-T-Te-Te		this study
NP_794446	C_starter_-A-T-^L^C_L_-A-T-C/E-A-T-^L^C_L_-A-T-Te	amphibactin -like?	this study
NP_794271	PKS-T-C?-A-T	unknown	this study
NP_794272	^L^C_L_-A-T-^L^C_L_-A-T-^L^C_L_-A-T-Te		

For each gene, the RefSeq identifier of the corresponding protein is given, as well as the modular organization of this protein.

A: adenylation domain, C: condensation domain, E: epimerization domain, T: thiolation domain, Te: thioesterase domain, Cy : cyclization domain, PKS : domain(s) belonging to the PolyKetide Synthesis.

The different types of C- and E-domains are mentioned as identified by the weblogo signatures.

The first cluster of genes (Cluster 1 in Table 2) is well identified and annotated. It belongs to the biosynthesis pathway leading to the production of yersiniabactin siderophore when the strain grows in an iron-limited environment [46]. Both NRPSs in this cluster are similar to the HMWP1 and HMWP2 proteins encoded by *ipr*1 and *ipr*2 genes in *Yersinia pestis* [48].

**Figure 6:**
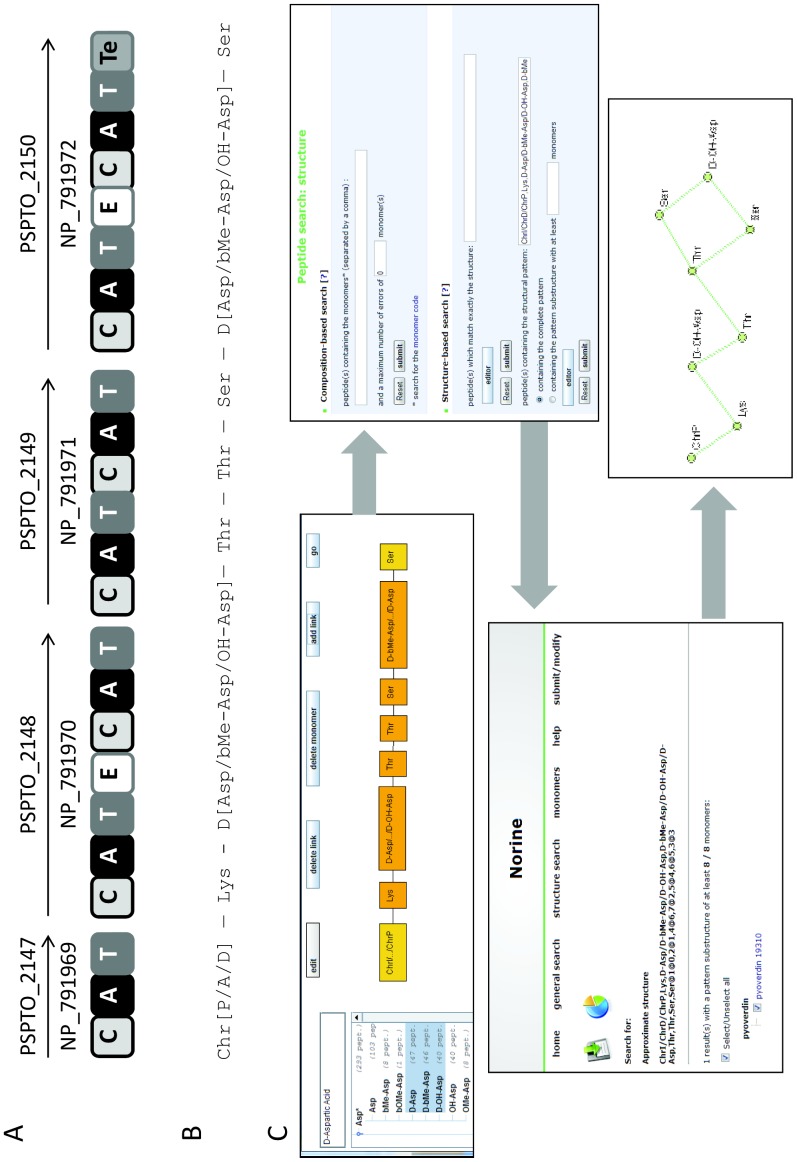
Biosynthesis of pyoverdin 19310 by *Ps. syringae* pv. *tomato* DC3000 : from the genomic cluster to the product. **A**: Organization of the synthetase in catalytic domains. The gene tags are above and protein id are below the arrows, A : adenylation domain, C : condensation domain, T : thiolation domain, E : epimerization domain, Te : thioesterase domain. **B**: Monomeric representation of probable peptides, when several monomers can occupy one position, they are indicated in brackets, the abbreviation of the monomers are those found in the Norine database. **C**: Successive screen prints of the process leading to the identification of pyoverdin 19310 using Norine.

The second cluster contains genes encoding proteins annotated as parts of a pyoverdin synthetase (Table 2). One protein is responsible for the synthesis of the chromophore moiety (NP_791957) and the four others direct side-chain biosynthesis (NP_791969 to NP_791972). In fact, the strain *Ps. syringae* pv. *tomato* DC3000 is known to produce a pyoverdin [46] but the structure of this siderophore has not yet been elucidated. We therefore applied the Florine workflow to get candidate structures for the NRP built up by the four proteins encoded in cluster 2. The architecture of the complete NRPS was defined by considering that the assembly line to be organized in the order of the genes along the chromosome (Step 1, Fig. 6A). Then, the monomer specificity of A-domains was predicted and all the C- and E-domains were analyzed (Steps 2 and 3, Table 2 and Fig. 6B). Because of the presence of an E-domain in module 2 together with a ^D^C_L_-domain at the beginning of module 3, we assume that the monomer in position 2 is a D-isomer. Exactly the same reasoning was applied for the monomer in position 6. The monomer specificity for both A-domains in modules 2 and 6 was predicted to be an aspartate residue (Asp). This was consistent with our previous observation that the E- and ^D^C_L_ domains are used for incorporation of D-Asp in pyoverdins. In a final step, the predicted peptide was compared to the pyoverdins annotated in the Norine database using the structure search tool. More than 60 different pyoverdins are currently described in the Norine database, all of them displaying one chromophore (ChrP, ChrI, ChrD) linked to a peptide moiety ranging from 5 to 12 monomers. Because of the chromophore, the numbering of each monomer is incremented by one in the final peptide (Fig. 6C). The monomers chosen for designing the candidate peptide using the Norine’s editor tool were a chromophore in position 1 (ChrP or ChrI or ChrD), a Lys monomer in position 2, a D-Asp-derivative in positions 3 and 7 (D-Asp or D-bMeAsp or D-OH-Asp), a Thr monomer in positions 4 and 5 and Ser monomer in positions 6 and 8. The pyoverdin 19310 (Norine ID NOR00199) is identical to one of the combinations proposed. In conclusion, the Florine workflow lead to the identification of the pyoverdin likely produced by strain DC3000 as pyoverdin 19310, like in other strains of *Ps. syringae*. The predicted structure now needs to be confirmed by MS and NMR analysis. Using the Florine workflow, the same pyoverdin was also found to be potentially produced by *Ps. syringae* pathovars *phaseolicola* 1448A and *syringae* B728a (not shown).

The third cluster is composed of 2 genes (PSPTO_2829 and PSPTO_2830) encoding 2 proteins (NP_792633 and NP_792634) annotated SyfA and SyfB, including 3 and 5 modules, respectively. The synthetase starts with the first C-domain of SyfA and ends with a tandem of Te domains of SyfB. It has previously been described to produce the syringafactin lipopeptide [47]. The six forms of syringafactin were submitted by the authors to the Norine database (differing by fatty acid chain length and the monomer in position 7) but without the isomery of each monomer. The signatures described in this paper and the Florine workflow helped us to determine the most probable isomers of each monomer. WL signatures corresponding to dual C/E- and ^L^C_L_ domains were identified in the protein sequences (Table S2, Table 2). Dual C/E-domains were suspected in modules 2, 3, 4, 6 and 8 allowing epimerization of monomers tethered on preceding T-domains (Leu1, Leu2, Gln3, Thr5 and Leu7). This is quite different from what was first suggested by Berti in the publication because they did not consider that the epimerization was occurring on the monomer preceding the dual C/E domain [47]. A comment about the predicted isomery of the monomers of syringafactins has been added to update data in the Norine database (NOR01075-80). The prediction was compared to the one obtained by NapDos which did not return good results when entire proteins are introduced. Indeed, SyfA was predicted to contain only 2 domains for condensation, both identified as dual C/E-domains, with the C-starter remaining undetected. On the other hand, the five domains of interest found in SyfB were predicted to be ^L^C_L_ which is correct for only two of them.

The fourth cluster of strain DC3000 only contains one NRPS gene (PSPTO_4699). The protein (NP_794446) is annotated as NRPS terminal component and is organized into 4 modules. Because it starts with a C-domain and no E-domain has been identified, the WL signatures were searched within the four C-domains to define their type. Without any ambiguity, C1 is a C-starter type, C3 is a dual C/E domain and C2 and C4 are ^L^C_L_ domains (Table 2). This clearly indicates that the peptide is probably a lipopeptide, but one not belonging to the super-family of CLPs because no Te tandem is present at the end and because of its relatively small size. However the presence of C-starter and Te domains indicates that the synthetase is probably complete. Structural comparison with peptides of Norine underlined the similarity with amphibactins (NOR00402, NOR00720-26), a family of 8 lipopeptides with siderophore activity produced by a marine bacterium [49], also containing 4 amino acids in the peptidic chain.

Cluster 5 consists of 2 genes (PSPTO_4518 and PSPTO_4519) annotated as nonribosomal peptide synthetase initiating- and terminal- components. The first protein (NP_794271) includes one PKS domain and one NRPS {C-A-T} module. None of the WL signatures defined in this study was detected in the Down-Seq region of this C-domain. This can be explained by the hybrid PKS/NRPS organization of the protein. The C-domain directly following the PKS part has probably a new specificity activity correlated with specific signatures. The second protein (NP_794272) harbours three modules and ends with a Te domain. All three C-domains of this protein clearly display WL signatures corresponding to ^L^C_L_ type (Table 2). Together with a lack of E-domain, this indicates that the peptide probably does not contain any D-monomer. The predicted tetrapeptide includes an “X” because no reliable specificity was obtained after A-domain analysis in module 3. No structural similarity with known peptides has been found, even when lowering the number of identical monomers to 3 among the 4 of the pattern. However, at this stage, several issues remain unclear. For example we do not know if the biosynthesis is simple and linear, and if the biosynthesis pathway is complete because the identified genes could result from a partial horizontal gene transfer. Up to now, only a linear mode of synthesis is considered in the Florine workflow, implying a perfect co-linearity between NRPS assembly line and the order of the monomers within the peptide [50]. But other iterative and non-linear modes of synthesis exist and should also be taken into account in the future.

## Conclusion

By this example we have demonstrated the interest of our Florine workflow that includes guidelines for monomer isomery prediction and confrontation with already described NRPs stored in the Norine database. Indeed, in the single genome of *Ps. syringae* pv. *tomato* DC3000 we have confirmed the presence of genes for yersiniabactin production, defined the type of putative pyoverdin secreted, specified the isomery of the syringafactin monomers and identified two new putative NRPs. The same strategy can now be applied to any genomic data (complete or draft genome) for any microbial strain. Florine is complementary to other tools such as antiSMASH and NapDos and is helpful to extend in the structure prediction of NRPs, especially for putative isomery identification.

The example of *Ps. syringae pv. tomato* DC3000 is also interesting because it confirms the co-existence in Pseudomonads of at least two strategies for integrating D-monomers into NRPs. The classical way through E- and ^D^C_L_-domains is encountered in pyoverdin NRPS and the alternative way through dual C/E domains which seems to be mainly restricted to cyclic lipopeptides of Pseudomonads. Further exploration of newly sequenced microbial genomes may lead to the discovery of new strategies for NRP monomer epimerization as suggested by the recent example of lysobactin synthetase from *Lysobacter sp*. ATCC 53042. This example also shows that our analysis of C-domains which clearly separates the shared common PF00668 domain from a downstream differentiating region, is useful for classifying newly discovered C-domains according to the presence or absence of particular amino-acid signatures.

## Supporting Information

Figure S1 **Identification of WL signatures within C- and E-domains in bacitracin (BacA, Bac B and BacC), syringafactin (SyfA and SyfB) and kurstakin (KrsC) synthetases.** The type of C-domain predicted by the various WL signatures is mentioned on the right side and for bacitracin synthetase, the known functional sub-type appears in the description of each domain (first line). Color code : grey for C-starter, yellow for ^L^C_L_, green for ^D^C_L_, blue for E, purple for C/E.
(DOCX)

Table S1 **Listing of NRPSs added to update the dataset used in this study.**
(PDF)

Table S2 **Translation of the graphic weblogos into aa signatures for C- and E-domains.** At each position the majoritary amino acid(s) is in capital letter(s) and the alternative possibilities are mentioned between brackets. Color code : grey for C-starter, yellow for ^L^C_L_, green for ^D^C_L_, Blue for E, purple for C/E, no color for Ct.
(XLSX)
