# Supplementary material for: Prediction of Monomer Isomery in Florine: A Workflow Dedicated to Nonribosomal Peptide Discovery
Source: PLoS One. 2014 Jan 21;9(1):e85667. doi: 10.1371/journal.pone.0085667 (PMC3897469; doi:10.1371/journal.pone.0085667)
Supplement: Figure S1 — Identification of WL signatures within C- and E-domains in bacitracin (BacA, Bac B and BacC), syringafactin (SyfA and SyfB) and kurstakin (KrsC) synthetases. The type of C-domain predicted by the various WL signatures is mentioned on the right side and for bacitracin synthetase, the known functional sub-type appears in the description of each domain (first line). Color code : grey for C-starter, yellow for LCL, green for DCL, blue for E, purple for C/E. (DOCX) [file pone.0085667.s001.docx]

**Bacitracin synthetase**

>BacA_C_DOMAIN_6 1669..2099 LCL_domain

KEYYPTSPAQQRMYMLSMLENERGAYHIPMALLVEGRINAMQLENALKTFLQRHEILRTG

FEIQNNELIQKIYENVDFRLEYECLDASITDQHALMEITSRYCKESIKPFDLSRPPLMRA

KLIKIDDIRHILVINFHHIISDGVSQGILMNEILELYSNVPLPEVNVQYKDYVEWNHTFN

^L^C_L_

QSAAMKKQEAYWLDVYRDIPSKLDFPYDYKRHHIDTFEGSSVFLEMERELSDHIRKLAKH

NGTTLYTVMLSAYYVLLNKYTNQTDIVVGTAAAGRLHPDLQDVFGVFVNTLALRNEVDTS

**YSFKEFLQQTKERTIAAFDNSEYPFDDLIRKLNGVRESNRNPLFDTMFVLEDARMFTKQK**

**GDVKLSPIIFELDNAKFDMIFNVLDFEQKIVLNIEYSTSLFKDETIQKIAEDYFRILEEV**

**SENLDVALHQI**

>BacA_C_DOMAIN_9 2703..3123 LCL_domain

KPYYRLSSAQKRLYILSQTGSHVAYNMPFAMTLEGDFDIRRFENTLKNMVKRHESFRTSF

VMIDGEVMQQIEKEIDFQVAYSDIGKESAEEKIKSFIRPFHLEKAPLLRAEVVKLNEREH

LLMFDMHHIISDGVSTDIFIQELGALYEGKSLKPFHIQYKDYAEWENSHARSEELKRQEE

YWLKTYKGDIPVLDLPIDHKRPLTKSSEGDTVTAAIESETFRKLQHMAKENGVTMYMLLL

^L^C_L_

AGYTALLSKYTGQEDIIVGTPAAGRNHEDIQHLIGMFVNTLAIRNHPEGKKTFRDYLQEV

**KENTLQAYENQDYPFEELVEKVNIKRDMARNPLFDTMLVYHNTDVKPFEAEGLRSRLVEI**

**KRGISKFDITVTASEAADGLRLEVEYSTTLFNKERMERLSEHLISLLEQAADHPDIAINQ**

**I**

>BacA_E_DOMAIN_12 3742..4197 E_domain

VTGETELLPIQKRYFANNKEELDHFNQSFMLFRKDGYDENIVRTAFNKILEQHDALRMIY

EEKDGDIIQYNRGYRENLFDLDVYDVRGFDSQEEKVFELATGIQKKSSIRKGKLVHLGIF

RADEGDHLLIAIHHLVVDGVSWRILFEDFETLYLQALKGEPLDIGYKTDSYQEFARQLKK

YAQSRRLLKEREYWQKALEADVPFIPAEKLERDTFEHSATLSIRIGPDVTAKLLRNAFKA

E

YNTEINDILLTALIAAVRDITGENKLKVMMEGHGREDILDGVDITRTIGWFTTVYPVFID

**LGEEKEISQNIKMVKEALRKIPNKGIGYGVLKYMTEELQKIQTQAPLSFNYFGEMNNDMN**

**RKVFSQSPFSPGESIGGKIVRHCAIEMNAISLNGELTIYTTFNQDQYQTSTIEQLNQSFK**

**ENLEKIVDHCVDKEGSDMTPSDYGDVSLGLEELELI**

>BacA_C_DOMAIN_13 4207..4640 DCL_domain

EKIYPLANMQKGMLFHNAMDQTSGAYFQQIVIKLKGRVHPDILEESFHEIVKRHEILRAS

FEYEITAEPRQIIARDRKTPFTSIDLTGENRTRQHRFIETYLKEDQEKGFDLSSEALMRV

CLIKMSDESYRLIWSHHHILLDGWCLGIVLSELFSLYGKIMKGESRRLKEPKPYGDYIKW

LEKQDQEEAVAYWKDYLKGYESRSELPAFNRGATSEEYCGKEKVISFSKELTTKITRIAK

^D^C_L_

QHHVTINTVLQGIWGMILAKYKNTDEVVFGTVVSGREAPVDGIEEMVGLFIHTIPTRISF

**EGARSFKEVLKKTQAESIESNRYSYMNLSEIQVLSEMKRELITHVMAFQNYAFDEELFRS**

**QSGETGFELEGVHGKERTNYNFNLTGVLEDEQLKLKLTFNENVYDNTIIETLEKHIITVA**

**EQVAEDETQTLRDI**

>BacB_C_DOMAIN_1 73..494 LCL_domain

SGTYPLSREQKRMFILNQLDDSKTAYNMPLAVKINGEVQISRLEQAWKALIKRHESLRTS

FVMLDGEPVQKIEQEAEFRLEYSELGDQSIQEKISRFIKPFELEKAPLLRAEIVKVDEAE

HMMMVDMHHIISDGVSIGILMKEFADCCEGKELSPLAVQYKDYSEWQRDIEQQSRLKKQE

AYWLNTFRGDIPVLNMPLDFPRPKIRSFQGNRTVVELDQDTTKKLKTIAAKNGVTMYMLL

^L^C_L_

LAGYTILLSKYTGQEDIIVGSPIAGRPHADLNGTIGMFVGTLALRNRPKGNMTFSEYVQT

**VKNNTLKAYENQDYQFDALIEHLGLTHDMSRNPLFDTMFDLQHADDFASEAGGGHFETYD**

**IPFHVAKFDVSLTAFLHGDNLKFDFQYCTDLYKKETVERMAGHFLNVLKDAAHHPELALS**

**EI**

>BacB_C_DOMAIN_4 1104..1525 LCL_domain

KEYYPLSSAQKRLYILNQIEEGQTAYNMPFAMKIKGELQTDKAEKAFRTLIKRHESSRTS

FVTINGEPVQNINEEVTFEMKYRELDNCSLRERMNQFIRPFELEKAPLLRAELVRVNAAE

HILLLDMHHIISDGVSIGILMKEWAALYEEKELAPLKIQYKDYSEWQRDPWQKDRLKKQE

ESWLSVFQNDIPVLNMPTDFPRPQMQSYEGDRIAFAIERELTDKLKKTAKENGVTMYMLL

LAGYTILLSKYTGQEDIIVGSPIAGRTREELEQTVGMFVGTLAMRNHPKGGRTFIEYLQD

^L^C_L_

**VKENTFNAYENQDYPFDELVDKLDLERDISRNALFDTMFDMQALDDAEPDIEGLHVEPVD**

**LEFQISKFDLSLTAAESAGVITFHLEFCTRLYKKETAETLAQHFVNILRDISDHPQKTLN**

**DI**

>BacB_E_DOMAIN_7 2141..2598 E_domain

VQGQVPLTPVQRSFFEANQREQNHYNQAFMLYRENGFAERIVEKVFRKLTEHHDALRMVY

WEKNGDIIQHNRGLEDSVFDLYVYDLKTEKNLEKTVYQIATNIQKDISISEGKMIKLCVF

KTTEGDHLLIAIHHLLVDGVSWRILFEDFEAAYGQALQGKPIELGYKTDSYKTFSEKLAE

YANSKKLLKEQEYWREISKGKMAFLPKHRQAAHDNYENSRTLRISLSQTETEQLLKEAHK

E

AYNTQINDLLLTALLIASRQLTGENRLKILMEGHGRDDILQDVDITRTVGWFTAMYPVFI

**DLEDEADLSVMIKIVKETLRKIPNNGIGYGILKYLRKDEGLLKDEKPPILFNYLGELDHD**

**LTTEQFSSSKLSAGQSIGEKSARDASVEIDSVVAGRQLMISTTFNEYEYSPDTISELNQA**

**FKESLQMVISHCTGKHETEKTSSDYGYDKLSLEDLEEL**

>BacC_C_DOMAIN_1 6..433 DCL_domain

EKIYPLSNMQKGMLFHAMKDEASHAYFEQFIIELKGDVDERMFEESLNEVMKRHEILRAS

FHHRLDEPLHVIIKDRHMKFDYLDIRGRHDQDGVLERYLAEDRQKGFDLAKDTLMRACLI

RMSDDSYQFVWTYHHILLDGWCLGIILDELLTIYEMKRKGQNHQLEDPRPYSDYIKWLED

QDKEEAQSYWESYLSGYDQKNSLPKLRTPSETGFKRREKTIECSKELTNRLIKLANRNHV

TINTVLQSIWGVILAKYNNSEDVVFGTVVSGRDAEVEGIETMVGVFINTIPTRIRLDKDK

^D^C_L_

**LFKDVLRQTQADALESSRYNYMNLAEVQALSELKNDLIDHVMVFENYAVDQKAFEEKNDV**

**GFEMVNVSGEEQTNYHFSISAALDDQLKLLFIYDENVYDTTIIETLEKHIITVAEQVAED**

**ETQTLRDI**

>BacC_C_DOMAIN_4 1049..1470 LCL_domain

KEYYRLSSAQKRLYILDQIEGSGLSYNIPFTMKVKGRFDIRRFENALKTIIQRHEALRTS

FLMADGEPVQKIEKEVDFSIKCSKIQSLSIQEIIKQFVRPFDLKKAPLFRTEVVKVDDEE

HIILFDMHHIISDGASMGVLTKEICDLYGGKELKPLSLQYKDYSEWQRDFYQKDEMKRQK

EYWLNIFKGEIPVLNMPTDYPRPQMHSVEGDRIGFAIDGELTKKLKRIAKDNGATMYMLL

^L^C_L_

LAAYTVLLRTYSGQEDVIIGTPIQGRKHHELKHVIGMFVNTLAMRNHPKGDKTFAEYLQD

**VKETALKAYENQDYQFDDLVEQLDLERDMSRNPLFDTMFVLQNLEKADAEIEGLTFEPFE**

**SDIHISKFDLTLSAIEKDSKIEFDLEYCTKLFKRETVERMAAHFVRVLEDISKRTDKRLD**

**QI**

>BacC_E_DOMAIN_7 2076..2533 E_domain

VTGHAELTPIQKWYFANNKEELDHFNQSFVLFRKGGFDESCVKKAFNKIMEQHDALRMIY

EEKGGDFIQYNRSFREDLFDLDVYDVRGLDRQAEKVYELATSIQKLSSIRKGKLVHLGIF

RADEGDHLLIVIHHLVVDGVSWRILFEDFETLYSQALKGQTLEIGYKTDSYQEFARRLKA

YAHSRTLSKEAEYWRNIAKARVRFIPPKNVLKEDVYENSTTLSIKLGKEATADLLRNTNR

AYNTEINDILLTALLTGARDITGENKLKVMMEGHGREDILEGVDITRTIGWFTTMYPVLL

**DAGEEKALSQQIKMVKETLRKIPNKGIGYGLLKYMAEDPDFTNEEKARISFNYLGDIDAD**

E

**MNRGEFSGSSFSEGESIGGKIARSHSIEINAIVMNHELVIHTTFNQMEYEKDTISRLNHQ**

**LKERLEQIIKHCTQQTESERTPSDYGDTNISLAELEEI**

>BacC_C_DOMAIN_8 2542..2974 DCL_domain

EKIYPLANMQKGMLFHAIEDHTSDAYFQQTVMDIEGYVDPAILEASFNDIMKRHEILRAS

YEYEIVEEPRQIIIENRSIDFTYFNIAKSSAQQQEMFIERLLNEDRKKGFDLSKDVLMRA

YLLKTAERSYRLVWSHHHILLDGWCLGIIMRELFVIYENRMNGKASPLKETKPYSDYIKW

LERQDQEEARQYWREYLKGYEEQAQLPTLTKRKKSSRYDRREKVIHLSKQLTKQLKELAA

^D^C_L_

KNSVTLHTVIQTIWGLMLTRYTKIDDVVFGTVVSGREANVDGIEDMIGLFINTIPTRIRF

**NEQARFNDCLQKVQEDAIQSNRYNYMNLAEVQALSSLKKDLIDHILVFENYEADEQDFEE**

**SQMKTGFKVNEISAAEQSITAFSMSVTPGEELTLVLTYDGNVYDRDIINNIEGHIKRVAE**

**QVTANENRKIAEI**

>BacC_C_DOMAIN_11 3585..4006 LCL_domain

KEYYALSSAQRRLYILNQIEPGGLSYNMPFAMKIAGDFDVDRFEDAFRQLIERHEALRTA

FVMVDGEPVQKIEKEVDFKVKYGRLGQDPLEEKIKAFIKPFALEKAPLLRAEVLKASGDE

HVLMLDMHHIISDGVSMAIFTRELAELYEGKTLPPLTIQYKDFSEWQKLFYQKDEVKRQE

DYWLNVFQGEVPVLNLPADEKRPQKRSIEGDIVQFEIDGETSAMLNKLAKENGATMYMLL

^L^C_L_

LAGYTTLLAKYTGQEDIVVGSPIAGRHHSDLKHVIGLFINTLAMRNHPKGDMPFADYLKE

**VKETALKAYENQDYPFDELVEKLDVKRDMSRHPLFDTMLVLQNFDGDEADIDGLTFQPLQ**

**TEVNISKFDLTLTAAETNEGIQCVFNYSTKLFKRSTIERMAGHLINILKEAANDPHMPLS**

**DV**

>BacC_C_DOMAIN_15 5089..5522 DCL_domain

EKIYPLANMQRGMLFHALEDKESQAYFEQMAINMKGLIDERLFAETFNDIMERHEILRAS

IEYEITDEPRNVIIKDRKINLDYHDLRKQSPAEREQVIQAYRKADREKGFRLNSEPLIRA

ALMRTEDDSYTFIWTNHHILLDGWSRGIIMGELFHMYHMKEARQKHRLEEARPYSDYIGW

LQQQDKEAAKAYWRNYLSGFTEKSPISVLAGSSGHAKYKRKEAVIEFPEQLTGRITELAS

RNNVTFHTVLQCIWGMLLARYNQTDDVVFGTVISGRDAQVTGIEKMVGLFINTVPTRIRL

**DKSQSFKELIKSVQEQALEGRTYHDMNLSEVQSLSELKRELLDHILIFENYAVDQSAFET**

^D^C_L_

**SGKRGAGFVFEEIHAEEQTNYGFNIVAVPGERLVIKLTYDGNIYHDHIIAGIKGHLQQVM**

**EQVVQHEDQSLNDI**

>BacC_E_DOMAIN_14 4622..5079 E_domain

VEGEVLLTPIQQEYFSLNKTDRNHYNHAVMLYRKNGFDESIVKRVFKEIIKHHDALRTVF

TEEDGKIIQYNRGPDKQLFDLFVYDVSSENDQPQKVYQLATELQQSIDIETGPLVKLAVF

KTNNGDHLLIIIHHLVVDGISWRILFEDLAIGYSQLANGEKVEFYPKTASYQAYARHIAE

YAKSVKLLSEKQYWLKAIAEGVEFLDMNENAGAFKVEDSRTFSTELEKEETKRLLRETNR

AYHTEINDILITALLVAARDMNGQNQLRITLEGHGREQVADGIDISRTVGWFTSKYPVFI

E

**DLGQETDMSRTIKMVKEHLRNVPNKGIGYGILKYLTRDSEIAKGAASPILFNYLGQLDED**

**INSGEFSSSHLSPGEAAGKGITREHPLEINAVVFRGKLAIQTTYNTRAYSEDVVRAFAQN**

**YKEALKAVIRHCAEREETEKTPSDYGDKGISLDQLEEI**

**Syringafactin synthetase**

>SyfA_C1

APTFSLTAAQRDIWLDQISRGDSPLYNIGGYLQITGPMDAQALQRALAQLVAAHEGLRTV

LMPGAGADGLPLQTYAASIPMPLAVHDFSDHLDPASSALALISEQMQRPCVFDGRPLVEF

CLIRLATDRYWLASQAHHLILDGWGFGQMMKSLGELYTAQVEGHSLELDAPRYSDFIVDD

VRYHASPRFAKDKAYWLDKYQNLPEPLLVSRYHNRRSTDPAPSHAWVQALPEALHARMKK

C-starter

FAESHNASTFHVLLAALHVYFTRTTQRKEWVVGLPLLNRTGAHFKATLGHFAQVSAVRMA

**FAEGLDFGALVIEVRDALKRDFRHQRFPLSELNRSLELSREERAQLFEVSVSYELEDHAY**

**RYGEAQAKTVKVSNGFEATPLAIHLRSNSLNDDASLHMVHHRAWIDDAEAQAIAGRLLHI**

**LEQGVESPALQIDDF**

>SyfA_C2

QEIYPLAPLQKGILYHHLTAGQGDPYLLQWRLAFDSLERLHAWAAALQQVINRHDILRTS

VVWEGLESPQQVVWRRAELTLQAVDFEAEDSQISVIDRLQRRYDARSHRLDLAQAPLMRL

VHARDSSSGETVAILLFHHLVLDNTAMEVVSREMQALLSRQYSALKTPVPYRNYVAHVNL

C/E

RNDDACHKAFFSEMLADVEEPTLPFGIHDVPADGSGIEEDRRTLDNDLALRLREQARQLG

VSAASLMHVAWARVLSVLANRRDVVFGTVLLGRMSGEGAERALGVFINTLPLRVDTALAT

**RAAVKAVHSRLAALIAHEQASLVLAQGCSGVASGTPLFSALLNYRHSAEVKPHDGEGLWQ**

**GVRVLGGDVRSNYPLTLSVDDLGENFDLHVLAMQGMGAERVAGWMQNTLEQLVQALERAW**

**PLA**

>SyfA_C3

QEIYPLAPLQEGILYHHLTAEQGDPYLLQLRINVDSLDRLNAVAAALRTVIARHDSLRTA

IVWQGLEVPQQVVWRHADLTVEHVASAQIDAEPGAARMDLARAPLIRLLYSPDATGAGLS

ATLQFHHIVVDATALEVMREEMLAHLRGEPGPTLPAVPYRNYVAQARLGVSEAEHEAYFR

C/E

EQLGDIDAPTLPFDLRDVQGDSRTIEEARQVIPDALLRGLRSQARQLGVSVASLLHLAWG

RVLAAATGNPRVVFGTVLLGRLQGGAGADRGMGMFINTLPLRIDLDNVGVRDGARATHAR

**LAQLLGHEHASLAQAQRWSGVAAPLPLFSAILNYRHAAGQARQDAQHDAWQGLDILASEK**

**HTNYPLSLNVDDLGDSLRLSVTVPSEIGARRICGYVQQVLAGMLDALENQPDLPLQRL**

>SyfB_C4

QDLYPLAPLQQGILYHHVTATQGDPYVMQVQFAFSDQPRLEAFAQALQTVINRHDILRTS

VHWDGLDTPVQVVWRHAELKVDSVSTGAGVTMDLGQAPLMRLVCHESAPADGHGVKATLL

FHHIAMDHSALEVVRHEIQACLLGQANALGVPVPFRNYVGQALLGVSEEEHETFFREMLA

DLDEPTLAYGLQDLSGDGDDLEEHSITLDLLLCQRLRAQARTLGVSVASLFHLGWAQVLA

C/E

GLTGQPRVVFGTVLMGRLLGAEATERALGIFINTLPLRLDLDDQDVRGAVRTTHQRL**TAL**

**MRHEHAPLALAQRCSGVQAPTPLFNALLNYRHSAPAQASGETWQGIEVLQAQERSNYPLV**

**LSVDDLGEAFGFTAQTSAGIEPQRICAYLQRAMESVIDALEQTPQMPVAQL**

>SyfB_C5

NGPVPLSSAQQRIWFMAQMEDANSAYNISLGLKLTGPLDSRALKRALERIVARHDSLRSR

FSQEDDIAWVQAAPITSVPDICWQDLRGHGADALRAVAQEEAAQPFDLRHDLPVRGRLLC

LAEERHVLLLTVHHIVADGWSLGVLTRELTALYQAFSQGLADPLPPLTLQYGDYAVWQRT

WLDAERLSHQADYWQQALTGAPVLLTLPTDRPRPAHQDYSGASVALTLDARLSTDIRTFC

QAQSVTPFMLFMGAWAVLLSRLSGQEEVVVGMPVANRRRAEIEGLIGLFVNTLAVRVDTS

^L^C_L_

**GEPDAVTLLARIKARVVAAQDHQDLPFEQVVERLRPPRSLAHSPLFQASLTWDGSQGLDL**

**QLGDLQLEPLDEQAAFAKFDLALSVGDSAEHFRCIVEYATALFDRSTVERYLGYLEAILR**

**GMVADGQTVVNHI**

>SyfB_C6

QDIYPLGPLQTGILYHHLTAGDRDPYLLQPQFAFADTSRLDAFCQALQRVIERNDILRTA

LCWEGLQAPVQVVWRQAPLRIQETPLPELFNAPRMELTQAPLLHLVYAHDPDNQRITAVL

RYHHVIMDHIALDVLSHELQAILLGNEAGLAAPVPYRNYIAHVLQGPGDDAHETFFREQL

GDVDEPTLPYGLAMTSAEQIPGEARLKLDSALCSQVRDQARQLSVSAATLMHLAWAQVLG

QLSGRDSVVFGTVLLGRLRGGEGGERALGVFINTLPLRMDLAGHSARSAVLDLHGRLVGM

C/E

**LAHEHAQLALVQRCSALPAGAPLFNTLLNYRHSAVSQVDDPASSAAWQGIAVIHAEERSN**

**YPLTVCIDDFGDDFGLTVQAAPGIDPQRICAYLQQALVHLVQALQQPPKTALIES**

>SyfB_C7

DQSLPLSFAQQRLWFLAQLEGGSEAYNISLALSLRGPLDVNALTAALARIVERHETLRSR

FIACEEGAEVFFAELPGSSLLHVEDLRLCPETLAERVVSEAATPFDLTRGPLIRGSLLQL

EDERHVLLLTVHHIVADGWSMGVLTRELLALYTALRHGKADPLPALAIQYADYAVWQQSW

MSGERLQHQAAYWRQTLDGAPTLLTLPTDRPRPAQQDFAGASLAVRLNSQLTAGLRALAQ

^L^C_L_

RQGVTLYMTLMTAWGALLACLSGQAEVVIGSPIAGRGRAELEGLIGLFVNTLAVRIDTSS

**AATGEALLAQVRTRVLEAQDHQDLPFEQVVEIVRPARSLAHAPLFQTTLNWLAGDSSLPQ**

**MDGLSLALVEQSAQTSKFDLSLNLGEHGDALVGTLDYATALFDDTTVQRYCGYFEQLLQA**

**LVNDQQTVLAQV**

>SyfB_C8

QDIYPLGPLQAGIFYHYLSAGDDDPYRLQARFAFADRSRLDAFCQALQQVIARNDVLRTS

LCWEGLETPVQVVWRHALLPVIELPLAALHDPEPLNLLDAPLLRLVHAEDPDNQRIVAVL

LFHHLIMDHVALDLLSHELQAVLLDQQAQLPAPVPYRNYIAHTLLGPGNDAHETFFREQL

GDLEEPTLPYAQATLPGPEVPGEARLRLDAALSQRIRNQVRQLGVSPASLMHLAWAQVLG

RLSGRDTVVFGTVLIGRLGGAEGAERTLGVFINTLPLRIDLAEQTARDAVLQTHRRLTGL

C/E

**LAHEHASLALAQRCSALPAGAPLFSALLNYRHSAAPGSHDKAASTAWQGIELLQTAERSS**

**YPLTLSVDDLGEVFDLTALTSAGIDARRVCAYLACAVESLLVALEHA**

Kurstakin synthetase KrsC

>C_DOMAIN_1 7..448

KEAIPLSYAQQRLWFMDRFNPNSSLYNIPTVWHLKGNWIPESLEKGFNRLIERHESLRTV

FKEIGEQPVQQIVEFLPRALPVRDYSQLSLEVKEKEVDSLIAREAQEPFDLMNGPLIRNQ

^L^C_L_

LVQLEKDEWLLLCTMHHIISDAWSIGIFMNELLAFYEEETGGNPAKLSSLSIQYADFAKW

QKEWLQGDVLNRQLTYWQEELSGELPILQLPVDRPRPVKQTYPGAAYHVIFPYKLLSQLK

DISRQEGSTLFMTLMAAYQSFLARYTGQTDILVGSPIANRNHKGVEGLIGFFVNTLVYRS

**DLSGTPTFREILNQTKKKALKAYEYQDIPFEKVVEAVQPERSMSHSPIFQTMFTLQNIKQ**

**ERLDLPDRSIEMVESNMSIAKFDLSLTAYEVEEGLFVSFEYNTDLFDSSTIARMAGHFEN**

**WLNEITHQPDESYTKLSMLSDT**

>C_DOMAIN_4 1069..1504

GEAIPLSYAQQRLWFIDQFTPNSALYNMPMVCRLTGNWLLEALETGWNRLIERHESLRTV

FHEVNGQPVQQIKPYVFQSILKTDLTMLSSEDQEEEVKRLIQQETEVPFDLTEGPLIRAS

ILHVGEEEWILLCTLHHIISDGWSIGILLEEWMAFYEKATDGKVAELEPLPVQYADFAQW

QKGWLKEEVLDQQLQYWREELSGDLPVLQLPMDRPRPAIQTHHGSTYTLVLPSTLHDKLN

ELSRKEGATLFMTLLAAYQSFLSRYTGQEDILVGSPIANRNYREIEGLIGFFVNTLVYRA

^L^C_L_

**NLSGKPTFQDVLYQVRQKALKAYEYQDIPFEKIVEVVQPERSTSHSPIFQTMFILQNMKQ**

**EFPVLSSRSIEMIESHSPIAKFDLSVMAAETEEGLLFTFEYNKDLFNATTIERMAGHFEK**

**WLHEVSHHPQNPFHDL**

>C_DOMAIN_7 2116..2547

PLSYAQQRLWFIDQFAPNSALYNMPMACRLTGNWLPEGLELGWNQLIERHESLRTVFHEE

EGHPVQQIQPYIFRPLPQMDLTKLSLEERERKLEQWIQTEVESPFDLEQGPLFRGKLIRI

SEEEWVLLCNMHHIISDGWSMEILLQEWMAFYENAIGEKQAELEPLPVQYADFAQWQREW

LKDEVLNQQLAYWKEELSGELPVLMLPMDRPRPPVQTHHGLTHNVLLSRSLLDKLNELSR

^L^C_L_

QEGATLFMTLLASYQSFLARYTGQTDIIVGSPIANRNYREIEGLIGFFINTLVYRADLSN

**APAFQELLSQVRIKALKAYEYQDVPFEKIVEVIQPERNTSHSPIFQTMFTLQNTRQELPE**

**LHGRNIKVMESNAPIAKFDLSLTAAEVEEGLLLTFVYRTDLFDSLTIESMAEHFGNWLNV**

**IVDNPDKSLAKL**

>E_DOMAIN_10 3159..3620

IIGETILTPIQQRFFAQNHPNPHHWNQSMFFRTKEKLDIGSLEKAARNLLLHHDALRLRY

ERLPNGAWKQWNEGIEEQLMLTVILLDEVPEADWYEVIQKEINIAQEGLNLHEGPLMRMV

YFDEGEKTGRLFWTIHHLAVDGVSWRILLEDLQTAYTQAVQGQKIQLPMKSTSFKEWSEK

LQHYAETGISKEVLHYWEQQAEQDVVILPVDGTISNPVSAVTEEITVVLNENETRMLLQE

E

TLSTHRVQINEVLLAALVQATAACTGQPILSVDLEGHGREEIIEDVDLSRTVGWFTSIYP

**VHLNITSANTSIAALKAVKEQVRKIPNKGVDYGVLRYMNATLCEQLSSQHTPSISFNYLG**

**QFDQMFSSDAIFMPENEFKRLDHAAGSKRSHVIDVIGVVTNGKLQFTWVYNVEQFAKSTI**

**QSIAQNMLYQLSRLIQPSDGESALTISDFAMANLNQVGLTNV**

>C_DOMAIN_11 3633..4061

TDLYPLSPLQEGMIFHTLHDQGNEHVAPYIVQLSSMIKGKMDIPTFEQAWKSVIQRHEIF

RTAFVWDEIEEPLQVVYENIPFKVNKEDWRTMTTKEIEEKRKVFLALDRKQAFQFDEAPL

MRVTVIQEGEEEYRIVWTHHHILLDGWSLPLVFKELLTVYQKRMNGEAVKLPKPSPYKKY

^D^C_L_

IQWLREQDKEQAEKFWREKLKGFTAPTLLGLESKEEEKGYTEKVTYLSEEQTQALQGWAK

**RNKFTLSTVIQGAWAYLMSRYSGENDIVFGVTSSGRPTEIIDVENIVGPFITTSPTRIQL**

**MDDIKVIDWLQKIQEEEIERRQYEYASLTEIQGWSEIPRGTPLFNCLYVFENYPVKEESS**

**GNLEIGELKGVEQTHYPLGLAVVPGIQLSLKLMYDRSKFNRVTIERMLGHLHKVLMQMLK**

**NIDQNLSEL**
