# Supplementary material for: Prediction of Monomer Isomery in Florine: A Workflow Dedicated to Nonribosomal Peptide Discovery
Source: PLoS One. 2014 Jan 21;9(1):e85667. doi: 10.1371/journal.pone.0085667 (PMC3897469; doi:10.1371/journal.pone.0085667)
Supplement: Table S1 — Listing of NRPSs added to update the dataset used in this study. (PDF) [file pone.0085667.s002.pdf]

Uniprot ID	Synthetase	Strain
**A5YBV1**	Fusaricidin synthetase	*Paenibacillus polymyxa*
**B1YQA7**	NRPS	*Burkholderia ambifaria* (strain MC40-6)
**B4E994**	Ornibactin synthetase	*Burkholderia cepacia* (strain J2315 / LMG16656)
**O68006**	Bacitracin synthase 1	*Bacillus licheniformis*
**O68007**	Bacitracin synthase 2	
**O68008**	Bacitracin synthase 3	
**P0C061**	Gramicidin S synthase 1	*Bacillus migulanus*
**P27206**	Surfactin synthase subunit 1	*Bacillus subtilis* (strain 168)
**P39845**	Plipastatin synthase subunit A	
**P39846**	Plipastatin synthase subunit B	
**P39847**	Plipastatin synthase subunit C	
**P94459**	Plipastatin synthase subunit D	
**Q04747**	Surfactin synthase subunit 2	
**Q0B1F5**	NRPS	*Burkholderia cepacia* (strain AMMD)
**Q0B1F6**	NRPS	
**Q0B1F7**	NRPS	
**P0C062**	Gramicidin S synthase 1	*Brevibacillus brevis*
**P09095**	Tyrocidin synthase 1	*Brevibacillus parabrevis*
**O30408**	Tyrocidin synthase 2	
**Q70LM4**	Linear gramicidin synthase subunit D	
**Q70LM5**	Linear gramicidin synthase subunit C	
**Q70LM6**	Linear gramicidin synthase subunit B	
**Q70LM7**	Linear gramicidin synthase subunit A	
**Q9R9I9**	Mycosubtilin syntase C	*Bacillus subtilis*
**Q9R9J0**	Mycosubtilin syntase B	
**Q9R9J1**	Mycosubtilin syntase A	
**A8MN36**	Putisolvin synthetase A	*Pseudomonas putida*
**A8MN37**	Putisolvin synthetase B	
**A8MN38**	Putisolvin synthetase C	
**Q0PH94**	Massetolide synthetase C	*Pseudomonas fluorescens*
**Q0PH95**	Massetolide synthetase B	
**Q0PH96**	Massetolide synthetase A	
**Q83VS0**	Syringopeptin synthetase C	*Pseudomonas syringae pv syringae*
**Q83VS1**	Syringopeptin synthetase B	
**Q9FDB3**	Syringopeptin synthetase A	
**Q09164**	Cyclosporin synthetase	*Tolypocladium inflatum*
**C3GUG5**	Kurstakin synthetase A	*Bacillus thuringiensis sv pondicheriensis*
**C3GUG6**	Kurstakin synthetase B	
**C3GUG7**	Kurstakin synthetase C	
**WP_003284246**	Kurstakin synthetase A	*Bacillus thuringiensis sv kurstaki*
**WP_000503035**	Kurstakin synthetase B	
**WP_003284247**	Kurstakin synthetase C	
